# Report of Committee on Drugs and Medicines

**Published:** 1869-07

**Authors:** N. S. Davis

**Affiliations:** Chicago, Ill.


ARTICLE XXVIII.

### REPORT OF COMMITTEE ON DRUGS AND MEDICINES.

READ TO THE ILL. STATE MEDICAL SOCIETY, JUNE 19, 1869.

BY N. S. DAVIS, M.D., etc., Chicago, Ill.

The constitution of the Society requires the Committee on "Drugs and Medicines" to report "on all the improvements and discoveries in Pharmacy and Materia Medica, effected during the year, and on all subjects connected with the sophistication and sale of drugs and medicines."

Two well defined duties are thus enjoined on the Committee. One to examine and report on the merits of such new remedial agents as may have been discovered or brought into notice during the year; and the other to notice the practice of mixing and adulteration of drugs. Following strictly the requirements under the first head we should have very little to report, for it is probable that the actual discoveries and improvements in the list of medicines during the past year have been few.

Still it may be profitable to call your attention to some articles, which if not new have been applied to new purposes, or used in new forms and combinations. For whatever improvements may have been made in Pharmacy during the past year, your Committee would refer members of the Society to the volume of Transactions of the American Pharmaceutical Association, recently published, and to be had in this city from E. H. Sargent, Druggist, corner of State and Randolph streets. It is a volume replete with useful information in reference to Pharmaceutical processes, inferior drugs, adulterations, drug laws and tariffs, etc., etc.

We do not think it proper to copy for re-publication extracts on these topics.

We will touch only on one subject connected with Pharmacy. During the last few years there has been a rapidly increasing tendency on the part of Pharmaceutists to manufacture and urge upon the public and the profession proprietory compounds, such as Elixirs, Concentrated Powders, Fluid Extracts, etc.

And in the opinion of your Committee the members of the profession have been too ready to adopt and use such preparations in practice. In the opinion of the Chairman of your Committee, this has been a prominent evil.

1st. The use of such compounds, instead of the officinal articles of the Pharmacopœia, destroys all uniformity in use of drugs by different practitioners, and makes it extremely difficult to compare the results of medication in the same forms of disease by different practitioners.

2nd. There is no security for the continued uniformity in the strength of such compounds.

3rd. The use of such proprietory compounds by the profession, gives to manufacturers and druggists greatly increased influence in imposing them on the community as *popular remedies.*

4th. Their use by physicians tends strongly to beget carelessness in prescribing, that is, carelessness in adjusting the exact proportion of each ingrediemt in a compound prescription, to the condition of individual patients.

Having at hand a compound of quinine, strychnine, iron, etc., it makes a strong inducement to give the same to A, B, and C, though, in fact, A needs only one grain of quinine to five of iron, and C needs two of quinine to two of iron. We hold that it is one of the most delicate and important duties of the physician to adjust the proportion of all compound prescriptions to the pathological condition of each patient. And his success in the cure of disease depends as much on his skill in this respect as on his selection of the remedies he shall use.

The true duty of the pharmaceutist and druggist is to furnish the profession with individual articles of the Materia Medica in their purest, most concentrated, and most convenient forms, leaving the physician to combine them to suit each of his patients. In relation to individual new remedies we will only allude briefly to the following:

### 1. Purified Opium—Svapnia.

An article bearing the above titles has been brought prominently to the notice of the profession by Dr. J. M. Bigelow, of Detroit; and manufactured by Frederick Stearns, of that city. It is claimed that this article consists of the morphia, narceia, and codeia of the opium, in their natural proportions and combinations; while the thebaine, pappaverine, narcotine, and woody fibre are entirely excluded.

Dr. Bigelow represents it as containing all the anodyne and soporific properties of opium, without the tendency to leave nausea and other unpleasant effects to the same extent as either the opium or morphia. If there is any advantage, however, to be derived from a combination of the morphia, narceia, and codeia, we see no reason why each physician should not combine them in just such proportions as his patient may need.

### 2. Sweet Quinine.

This is another article prepared by Dr. Bullock from the Peruvian bark, and is represented to possess all the medicinal properties of the sulphate of quinine, without its bitterness. Its chief recommendation is the greater ease of administration, especially to children. It appears to be simply the alkaloid quinia so mixed with the extract of liquorice that when given in water or simple syrup it has comparatively little bitterness. But if mixed with either acids or alkolies its bitterness is fully developed. It may be given for the same purposes, and in the same doses, that we use the sulphate of quinine.

### 3. Carbolic Acid.

The uses of Carbolic Acid as an external and local application are sufficiently familiar to the profession, but its internal administration has attracted but little attention until the past year. From the trials made with it thus far, it would seem that when given in moderate doses it was capable of relieving flatulency, arresting fermentation in the alimentary canal, and correcting offensive secretions. It has also been found to lessen the amount of lithates in the urine. It will probably be found valuable in certain cases of indigestion, and in some stages of typhoid fever and dysentery. As a local application in the form of spray, it is very valuable in correcting offensiveness of the secretions from the throat and nostrils in diphthentic, catarrhal, and syphilitic inflammations of those parts. One difficulty in prescribing carbolic acid is the uncertain strength of the preparations kept in the drug stores.

The crystals of carbolic acid deliquesce so rapidly when exposed to the air that druggists are often embarrassed in weighing them for use in prescriptions, and there is no recognized solution of uniform strength.

Although the crystals deliquesce rapidly into a liquid, yet water will hold in solution properly only a limited quantity of the acid either before or after deliquescence. It is very desirable that all druggists should keep a true saturated solution of known and uniform strength. Such a solution may be given internally in doses of from four to eight miniums.

4. Peroxide of Hydrogen.

The attention of the profession has been called to the medicinal properties of this article by Dr. B. W. Richardson, of London, and others, in such terms as to justify a passing notice.

It has been administered internally in diabetes and other diseases of debility or general cachexy.

It is supposed to be capable of increasing the amount of active oxygen in the system, and, consequently, promoting the oxydation of the tissues and the rapidity of organic changes.

If this supposition is true, it should constitute an efficient alterative and tonic.

Several cases of diabetes milatus are reported in the medical periodicals as having been cured by its use, and Dr. Richardson proposes it as a substitute for mercury and iodine in the treatment of constitutional forms of syphilis and scrofula. The liquid, as prepared by chemists, may be given in doses of from one to four fluid drachms, repeated three times a day.

5. Ozonic Ether.

This is another article that has been brought into notice during the last few months, chiefly as a remedy for diabetes. It is simply an ethereal solution of the *peroxide of hydrogen*, and consequently may be used for all the purposes for which that substance is applicable, as stated in the foregoing paragraph. The dose is from ten to thirty minims three or four times a day in water.

6. CASTANEA VESEA—CHESNUT LEAVES.

Dr. Nuziker, of Cincinnati, has recently published the statement that the infusion of the leaves of the Chesnut, drank freely, is capable of mitigating the severity and greatly lessening the duration of hooping-cough. He makes a simple infusion of the leaves, sweetens it with sugar or syrup, and allows the patient to use it as a drink.

It is worthy of further trial.

This list might be further extended, but not with much profit to members of this Society. While we should carefully avoid being seduced by every novelty in the way of new remedies, we should be equally careful to give all such as are based on any reasonable chemical or physiological grounds a fair and patient trial. For information concerning the adulteration, sophistication, etc., of drugs, and a variety of other kindred matters, your Committee would refer to the published Transactions of the American Pharmaceutical Association, which may be had of E. H. Sargent, druggist, of this city.

